# Mobile Apps for Suicide Prevention: Review of Virtual Stores and Literature

**DOI:** 10.2196/mhealth.8036

**Published:** 2017-10-10

**Authors:** Isabel de la Torre, Gema Castillo, Jon Arambarri, Miguel López-Coronado, Manuel A Franco

**Affiliations:** ^1^ Department of Signal Theory and Communications, and Telematics Engineering University of Valladolid Valladolid Spain; ^2^ VirtualWare Labs Foundation Bilbao Spain; ^3^ Psychiatry Service Hospital of Zamora Zamora Spain

**Keywords:** app, literature, prevention, suicide, virtual stores

## Abstract

**Background:**

The best manner to prevent suicide is to recognize suicidal signs and signals, and know how to respond to them.

**Objective:**

We aim to study the existing mobile apps for suicide prevention in the literature and the most commonly used virtual stores.

**Methods:**

Two reviews were carried out. The first was done by searching the most commonly used commercial app stores, which are iTunes and Google Play. The second was a review of mobile health (mHealth) apps in published articles within the last 10 years in the following 7 scientific databases: Science Direct, Medline, PsycINFO, Embase, The Cochrane Library, IEEE Xplore, and Google Scholar.

**Results:**

A total of 124 apps related to suicide were found in the cited virtual stores but only 20 apps were specifically designed for suicide prevention. All apps were free and most were designed for Android. Furthermore, 6 relevant papers were found in the indicated scientific databases; in these studies, some real experiences with physicians, caregivers, and families were described. The importance of these people in suicide prevention was indicated.

**Conclusions:**

The number of apps regarding suicide prevention is small, and there was little information available from literature searches, indicating that technology-based suicide prevention remains understudied. Many of the apps provided no interactive features. It is important to verify the accuracy of the results of different apps that are available on iOS and Android. The confidence generated by these apps can benefit end users, either by improving their health monitoring or simply to verify their body condition.

## Introduction

According to the World Health Organization, approximately one million people commit suicide each year, which is the second leading cause of death among people from 15 to 29 years of age [1]. There are also indications that for every adult who committed suicide, possibly more than 20 others attempted suicide [1]. Worldwide, suicides account for 50% of all violent deaths recorded among men and 71% among women [2]. In Spain, suicide causes twice as many deaths as traffic accidents and 70 times more deaths than gender violence [1].

It is important to highlight the fact that suicides are preventable; prevention efforts would require a comprehensive multi-sectorial prevention response in all countries. Based on the societal impacts that suicide has in many countries, it is very important that health services incorporate suicide prevention as a central component. In addition, alcohol consumption and mental disorders contribute to many suicides worldwide [2-4].

Early identification and effective management are key for people at risk for suicide, so they can receive the care they need [5]. Communities play a key role in suicide prevention: they can provide social support to the most vulnerable individuals, deal with their follow-up, fight against social stigmatization, and support those who have lost close relatives to suicide [6,7].

People feel anxiety and some situations can cause them to make decisions to end their life. Information and communications technologies (ICTs) can provide innovations to support the work of health professionals to address this issue. Mobile apps can also provide a valuable resource for communities and individuals by addressing social issues [3,4]. The aim of this article is to conduct a systematic review of existing mobile apps in Google and Apple online stores, and in the literature for suicide prevention. This research will help experts in the field and app developers gain insight into what mobile apps exist, and features that they can continue to research and develop.

Mobile health (mHealth) is a part of electronic health and refers to computers, smartphones, mobile health apps, and patient monitoring services and apps used for the purpose of positively increasing access to health information. For many health issues, especially those related to mental health prevention, remote patient monitoring is a good start and mobile apps on smartphones are an available means that can save costs and benefit hard-to-reach populations. However, it is important to verify the accuracy of the results of different apps that are available on the virtual stores, such as iOS [8] and Android [9]. The confidence generated by these apps can benefit end users, either by improving their health monitoring or simply to verify their body condition.

The goal of this paper is to present the state of the art of mobile apps designed to prevent suicide, in English and Spanish languages. This review of the scientific literature is presented to show scientific studies that demonstrate how such apps have been developed, and the results that have been obtained, when using mobile apps. This review allows us to promote the discussion of the results found, and to communicate the current state of these technologies to other members of the scientific community.

## Methods

### Search Strategy for the Reviews

Two reviews have been completed; the first was carried out by searching the most commonly used commercial apps stores, which are Google Play [9] and iTunes [8]. The second review was a search of published articles from the following scientific databases: Science Direct, Medline, PsycINFO, Embase, The Cochrane Library, IEEE Xplore, and Google Scholar.

**Figure 1:**
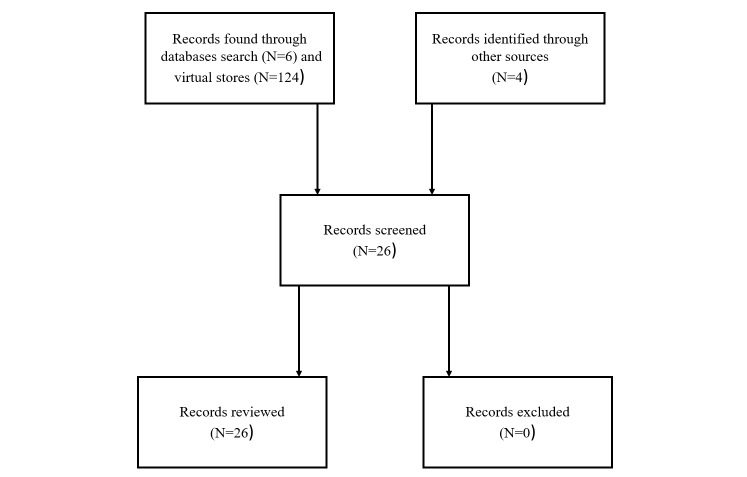
Flow chart of the steps in the review.

The search terms used for virtual stores were “*prevention*” and “*suicide*”. Only apps that focused on suicide prevention were studied; music, games, and other apps were dismissed. The literature review was developed on the indicated scientific databases. The combination of terms in titles/abstracts used in the literature revision was the following: “*prevention*” AND “*suicide*” AND “*app/mhealth*”. The results were limited to the last 10 years, from 2007 forward. Only articles published in English were studied. Figure 1 presents a flowchart with the steps that were followed in the review.

## Results

### Virtual Store Apps

A total of 20 relevant apps were found in the Google Play and Apple App Stores, although we note that some apps were available in both stores. A classification of these apps is shown in Multimedia Appendix 1. The characteristics chosen were: name, operating system, language, brief description, and price. The number of downloads has not been taken into account, because this value can change continuously. Only one app was found in Spanish (Prevensuic), which was designed by the Spanish Mental Health Foundation for the prevention of mental disorders and suicide. This app was developed for Android and iOS.

### Apps in the Literature

A total of 6 relevant papers were found in the online databases that were searched. Shand et al [10] evaluated the effectiveness of a self-help app for suicidal thoughts amongst young Indigenous people (with a total of 150 participants). This research was the first to evaluate the effectiveness of a self-help app for suicidal thoughts amongst young Indigenous people.

Aguirre et al [11] described mobile apps for one specific field of Human Service Organizations related to suicide prevention, and other topics in mental health and behavioral issues. This study analyzed 27 apps, and concluded that it was critical to carefully plan and develop the apps, and undertake ongoing evaluation of these apps [11]. Berrouiguet et al [12] proposed a mobile app for suicide prevention that was a connected tool used by the patient to describe his/her health status. The authors concluded that the app should be ergonomic, with data protection and question choices [12]. These facts should be considered in the future to increase acceptance by both patients and practitioners.

Larsen et al [13] found 123 apps referring to suicide, but not all were specifically related to suicide prevention. The authors warned that physicians should be wary in recommending apps [13]. McManama et al [14] evaluated a smartphone app intervention developed specifically for suicidal adolescents and their parents, named *Crisis Care*. Twenty adolescent-parent dyads participated in the pilot evaluation, and the results showed positive results for usability, acceptability, and utility of the app [14]. This app can be an adjunct to treatment for suicidal adolescents and their parents [14].

Kennard et al [15] showed the results from phase one of a treatment-development study for suicide prevention. This study used qualitative methods to collect information from families, teens, and physicians and evaluated acceptability and the feasibility to provide the intervention to patients and parents. All participants were constructive about the utility of technology in safety planning with the mHealth app, but limitations included privacy and confidentiality issues [15].

## Discussion

Suicide prevention is a health issue, and it is important that apps in this field are supported by professionals who make these tools as a way to care for their patients, and to prevent this situation that affects many countries.

Few commercial mobile apps aimed at suicide prevention exist, compared to other pathologies such as cardiology [16]. As far as existing literature is concerned, even fewer results have been found. Luxton et al [17] examined innovate apps related to suicide prevention. Some of these apps included gaming, text analysis, and virtual worlds, and the authors discussed the limitations and advantages of these technologies [17].

Marasinghe et al [18] conducted a randomized controlled trial examining whether a *Brief Mobile Treatment* intervention could improve outcomes related to typical care among people with suicidal behavior. Sixty-eight participants were recruited from a Sri Lankan hospital; people who received the intervention were found to accomplish important improvements in reducing suicidal ideation [18]. Larsen et al [19] showed an overview of different technological developments for suicide prevention. Some of these developments included automatic detection of suicide cases from social media and crisis detection from acoustic variability, among others [19]. The authors presented the mHealth app for Indigenous populations that was detailed in Shand et al [10]. Barriers to help-seeking included shame, feared loss of autonomy, and negative attitudes towards health care providers [10]. The use of mobile devices and apps continues to rise amongst young people, thus presenting opportunities to utilize these aids in overcoming help-seeking barriers.

Based on our experience in clinical and real cases, we conclude that mHealth apps are an essential tool that can help us to prevent suicide, considering the family, caregivers, and health professionals that are part of helping to save a life through the use of ICTs. Therefore, we consider it fundamental that clinical support provides validity to the apps offered on the Internet. The bibliographic review allowed us to examine the apps based on scientific studies from real cases that generated proven results.

As future work, we will develop and evaluate a suicide prevention app by looking at the shortcomings detected in the apps that were analyzed in this work. Saving even a single life via a mobile app is a breakthrough.

## Appendix

Multimedia Appendix 1 Suicide prevention apps in virtual stores.

## Abbreviations

ICT: information and communications technology
mHealth: mobile health
