# Supplementary material for: Mobile Apps for Suicide Prevention: Review of Virtual Stores and Literature
Source: JMIR Mhealth Uhealth. 2017 Oct 10;5(10):e130. doi: 10.2196/mhealth.8036 (PMC5654733; doi:10.2196/mhealth.8036)
Supplement: Multimedia Appendix 1 [file mhealth_v5i10e130_app1.pdf]

Multimedia Appendix 1. Suicide prevention apps in virtual stores.

Name	Supported<br><br>Operating System/s	Brief description	Language	Price
DMHS Suicide Prevention	Android<br><br>iOS	Provide information about suicide intervention, suicide prevention and mental health resources. It also provides direct link to a 24-hour telephone support Crisis Access Linkage Line (CALL)	English	Free
Suicide Prevention Help Squads	Android	Contains help plan or planning, treatment, and how to prevent the suicide	English	Free
Prevent Suicide - NE Scotland	Android<br><br>iOS	Aimed at users in the north east of Scotland. It provides helpful info for those affected by suicide	English	Free
HELP Prevent Suicide	Android	It is based on the Healthy Education for Life Program (HELP). It includes a list of warning signs, information on national and Oklahoma-specific resources, and steps on how to talk with someone.	English	Free
Ask & Prevent Suicide	Android	This suicide prevention information was collected by @StopTXSuicides - Mental Health America of Texas, under the Texas Youth Suicide Prevention Project, funded by the Texas Department of State Health Services and SAMHSA.	English	Free
Suicide? Help!	Android<br><br>iOS	Suicide? Help! - Tablet - An information app for people who are thinking about suicide or are worried about someone else.<br><br>As well as providing detailed information about suicide, such as how to get help and what signs to look for in others, this app also provides details of services the user can contact particularly in the UK and Tayside.	English	Free
Prevensuic	Android<br><br>iOS	It has a series of tips, a record of reasons for living and photos that evoke memories of positive moments and a list of warning signs that the user can complete to identify a state of suicidal ideation and be able to act on it as soon as possible. Prevensuic	Spanish	Free

		provides information to family members, people with some suicidal characteristics, and professionals. It is not clear the interaction of people with suicidal intentions and professionals. It is an app to provide general information about suicide in Spanish.		
Not Even One	Android	This resource app is designed for teens to help educate around a difficult topic - suicide prevention. This app will provide tips on how to talk about suicide, where to find resources and what to do when a crisis situation is encountered. Not Even One will provide educational information to help teens learn more about suicide prevention awareness.	English	Free
Stay Alive	Android	This app is a pocket suicide prevention resource, packed full of useful information to help you stay safe. You can use it if you are having thoughts of suicide or if you are concerned about someone else who may be considering suicide.<br><br>In addition to the resources, the app includes a safety plan, customisable reasons for living, and a life box where you can store photos that are important to you.	English	Free
Suicide Helplines in India	Android	Directory of National and Local Suicide Prevention / Crisis Helplines from all parts of India	English	Free
Suicide Preventive	Android	This suicide prevention application is aimed at professionals who are familiar with the "diagnosis and treatment of suicidal behavior" and come into contact with patients who exhibit suicidal behavior	Holandes	Free
Suicide Lifeguard	Android<br><br>iOS	Suicide Lifeguard is app intended for anyone concerned that someone they know may be thinking of suicide This suicide prevention information was produced by the Missouri Suicide Prevention Project, a joint effort between the Missouri Institute of Mental Health at the University of Missouri-St. Louis and the Missouri Department of Mental Health.	English / Spanish speakers	Free
Suicide Thoughts	Android	This APP will help you access a suicide threat and hopefully diffuse the situation. It will focus on pointing the person to their proper identity and purpose.	English	Free

Suicide Safety Plan	iOS	Suicidal Thoughts can seem like they will last forever – but these thoughts and feelings pass with time. This app is designed to support those dealing with suicidal thoughts and help prevent suicide.	English	Free
A Friend Asks	Android	It contains information about warning signs of suicidal ideation, how to get help, what to do and what not to do, the B1 Program and how to help a friend	English	Free
Virtual Hope Box	Android<br><br>iOS	The Virtual Hope Box (VHB) is a smartphone application designed for use by patients and their behavioral health providers as an accessory to treatment. The VHB contains simple tools to help patients with coping, relaxation, distraction, and positive thinking. Patients and providers can work together to personalize the VHB content on the patient's own smartphone according to the patient's specific needs. The patient can then use the VHB away from clinic, continuing to add or change content as needed.	English, Spanish, German, Simplified Chinese, Japanese, Polish	Free
Operation Reach Out	Android	It aimed to prevent suicide among military personnel	English	Free
We Care	iOS	This app is to support the Suicide Prevention/Sexual Assault Campaigns and serves as an educational and resource tool to reduce high risk behaviors with the goal of eliminating sexual harassment and sexual assault. By Army Training Support Center	English	Free
Suicide or Survive	Android<br><br>iOS	Some of its characteristics are: Tracking device for your mental wellbeing over time, mindfulness diary and timer and Feed your wolf of hope, between others.	English	Free
R U Suicidal	Android	R U Suicidal? is a free app designed to help someone who may be or is suicidal. It is predominantly a video of a Psychologist speaking directly to the user and aims to be warm, supportive and encouraging.	English	Free
